# GeneMANIA: a real-time multiple association network integration algorithm for predicting gene function

**DOI:** 10.1186/gb-2008-9-s1-s4

**Published:** 2008-06-27

**Authors:** Sara Mostafavi, Debajyoti Ray, David Warde-Farley, Chris Grouios, Quaid Morris

**Affiliations:** 1 Department of Computer Science, University of Toronto, King's College Road, Toronto, ON, M5S 3G4, Canada; 2 Gatsby Computational Neuroscience Unit, London WC1N 3AR, UK; 3 Department of Molecular and Medical Genetics, University of Toronto, King's College Road, Toronto, ON, M5S 1A8, Canada; 4 Banting and Best Department of Medical Research, University of Toronto, College Street, Toronto, ON, M5G 1L6, Canada

## Abstract

**Background:**

Most successful computational approaches for protein function prediction integrate multiple genomics and proteomics data sources to make inferences about the function of unknown proteins. The most accurate of these algorithms have long running times, making them unsuitable for real-time protein function prediction in large genomes. As a result, the predictions of these algorithms are stored in static databases that can easily become outdated. We propose a new algorithm, GeneMANIA, that is as accurate as the leading methods, while capable of predicting protein function in real-time.

**Results:**

We use a fast heuristic algorithm, derived from ridge regression, to integrate multiple functional association networks and predict gene function from a single process-specific network using label propagation. Our algorithm is efficient enough to be deployed on a modern webserver and is as accurate as, or more so than, the leading methods on the MouseFunc I benchmark and a new yeast function prediction benchmark; it is robust to redundant and irrelevant data and requires, on average, less than ten seconds of computation time on tasks from these benchmarks.

**Conclusion:**

GeneMANIA is fast enough to predict gene function on-the-fly while achieving state-of-the-art accuracy. A prototype version of a GeneMANIA-based webserver is available at .

## Introduction

The vast amount and increasing variety of genomic and proteomic data generated for model organisms creates an opportunity for *in silico *prediction of gene function through extrapolation of the functional properties of known genes. Genes with similar patterns of expression [1], synthetic lethality [2], or chemical sensitivity [3] often have similar functions. Additionally, function tends to be shared among genes whose gene products interact physically [4], are part of the same complex [5], or have similar three-dimensional structures [6]. Computational analyses have also revealed shared function among genes with similar phylogenetic profiles [7] or with shared protein domains [8]. More accurate predictions can be made by combining multiple heterogeneous sources of genomic and proteomic data [9]. Collectively, these observations have led to functional categorization of a number of previously uncharacterized genes using the so-called 'guilt-by-association' principle [10-12].

Algorithms that predict gene function using the guilt-by-association principle do so by extending a 'seed list' of genes known to have the given function by adding other genes highly associated with the seed list in one or more genomic and proteomic data sources. These algorithms typically compute a 'functional association network' to represent each dataset; in this network the nodes correspond to genes or proteins and the undirected links (or edges) are weighted according to evidence of co-functionality implied by the data source. Types of functional association networks include kernels used by support vector machines (SVMs) [9], functional linkage networks [13], and protein-protein linkage maps [14]. Individual functional association networks are often combined to generate a composite functional association network that summarizes all of the evidence of co-functionality. This network is then used as input to an algorithm that scores each gene based on its proximity to the genes in the seed list. When employed on multiple complementary data sources, these algorithms can accurately predict previously annotated gene functions in blind tests [15], suggesting that their predictions for unannotated genes are also quite accurate.

Despite these successes, guilt-by-association algorithms have yet to achieve widespread use in gene annotation or as sources of new hypotheses about gene function; to do so, their predictions need to become more accessible, more accurate, and more regularly updated. In principle, all available data should be used when generating hypotheses about gene function; however, compiling a large number of heterogeneous data sources, generating functional association networks to represent these sources, and then mapping gene identifiers among the networks is a complex and onerous task that is best handled by specialists. Centrally managed web-based 'prediction servers' are an efficient strategy to ensure that casual users have access to the best available predictions.

However, maintaining accurate and up-to-date prediction servers can be computationally prohibitive. Though a large number of algorithms have been developed to predict the function of unannotated genes by combining heterogeneous data sources (see [16] for a recent review), the most accurate of these algorithms have long running times, which can range from minutes [17] to hours [9] on yeast. Larger mammalian genomes increase the run time of these algorithms even more. As such, these algorithms cannot feasibly be run online and instead their predictions are made offline based on sets of pre-defined seed lists derived, for example, from Gene Ontology (GO) annotations [18]. However, because new data and annotations are being generated at a rapid rate, maintaining an up-to-date database of the best available predictions for all possible functions requires substantial and potentially unavailable computational resources.

Due to this limitation, most prediction servers sacrifice accuracy for speed by relying on a single, or a small number of, pre-computed composite functional association networks and using simple heuristics to score genes based on a given seed list (for example, see [13,14,19]). While the scoring heuristics are fast enough to provide online predictions for arbitrary seed lists, we will show that their predictions are much less accurate than more advanced methods. Furthermore, by using a single pre-computed network, these servers do not take advantage of the fact that different data sources are more relevant for different categories of gene function [2,9] and are not extensible to new or user-supplied data sources.

Here we demonstrate that it is not necessary to surrender either accuracy nor flexibility when building a prediction server by showing that GeneMANIA (Multiple Association Network Integration Algorithm) can, in seconds, generate genome-wide predictions that achieve state-of-the-art accuracy on arbitrary seed gene lists without relying on a pre-specified association network. We have achieved this goal through a series of algorithmic and technical advances that we have encapsulated in a new software package. With GeneMANIA, it is no longer necessary to maintain lists of *in silico *predictions of gene function because they can be recomputed as needed.

## Background

### Automated methods for predicting gene and protein function

Many algorithms for predicting gene function use a functional association network as a common representation of evidence of co-functionality for heterogeneous datasets. In this representation, nodes represent genes or proteins and the undirected edges (or links) between these nodes are weighted according to the evidence for co-functionality of the genes implied by the particular datasets. There are various techniques for deriving these weights depending on the source of data and it is still unclear how best to extract evidence of co-functionality (see, for example, [20,21]); as such we here use a single method to calculate these weights (see Materials and methods for details). Here, we assume that all of the association weights are positive.

### Predicting gene function from functional association networks

Following Lanckriet and coworkers [9], we pose the problem of predicting gene function as a binary classification problem and attempt to solve the problem of integrating multiple heterogeneous input data sources by assigning each functional association network derived from these data sources a positive weight that reflects its usefulness in predicting a given function of interest. Once these weights are calculated, we construct a function-specific association network by taking the weighted average of the individual association networks. In contrast to prior work [9,17] in which the weights and discriminant values were optimized simultaneously using the same objective function, we use a separate objective function to fit the weights, leading to a simpler optimization problem and a substantial reduction in run time.

We predict gene function from the composite network using a variation of the Gaussian field label propagation algorithm [22,23] that we adopted to be more suitable for unbalanced classification problems like gene function prediction. Label propagation algorithms, like most function prediction algorithms, assign a score to each node in the network, called the 'discriminant value', that reflects the computed degree of association that the node has to the seed list defining the given function. This value can be thresholded to make predictions. While there are a large variety of label propagation algorithms (for example, those based on functional flow [24,25] or Markov random fields [26]), our interest in the Gaussian field algorithm stems from the fact that it has a well-defined solution, it is derived from a principled framework, and it has recently been shown to perform as well as SVMs on gene function prediction tasks [17]. SVMs had previously been shown to be among the best algorithms for predicting gene function [27,28].

## Results

The GeneMANIA algorithm consists of two parts: an algorithm, based on linear regression, for calculating a single, composite functional association network from multiple networks derived from different genomic or proteomic data sources; and a label propagation algorithm for predicting gene function given this composite network. Below we describe the two parts of the GeneMANIA algorithm individually. We evaluate GeneMANIA on yeast benchmarks as well as the MouseFunc I benchmark.

### GeneMANIA label propagation

Gaussian field label propagation algorithms for binary classification take as input an association network, a list of nodes with positive labels, possibly a list of nodes with negative labels, and initial label bias values. For gene function prediction, each gene is associated with a single node and nodes representing genes in the seed list (that is, positive genes) are assigned an initial label bias value of +1, and those representing genes that are deemed negative examples are assigned an initial bias value of -1. In the Results section, we explore a couple of strategies to defining negative examples; here we will assume that these examples are provided. GeneMANIA label propagation differs from [22] in how we assign the label bias to unlabeled genes, which are those that appear neither on the seed list nor among the negative examples.

Given this input, label propagation algorithms assign a discriminant value to each node. These discriminant values are determined by letting the initial label bias of nodes propagate through the association network to nearby nodes. To account for potential noise in the initial labelings, GeneMANIA label propagation, like [22], allows the discriminant values assigned to positively and negatively labeled nodes to deviate from their initial biases.

Our label propagation algorithm assigns discriminant values by finding those that minimize a cost function that penalizes both differences between the discriminant values of neighboring nodes in the network as well as differences between the discriminant values of nodes and their label bias (see Materials and methods for details). This cost function allows information about the node labels to propagate through the network to affect the discriminant values of genes that are not directly connected to the seed list. As such, the initial bias values of labeled and unlabeled nodes are important in determining their discriminant values and those of their neighbors; to account for the fact that only a small portion of the genes are expected to be labeled as positive, in the GeneMANIA algorithm we set the initial bias of unlabeled nodes to be the average bias of the labeled nodes: $\frac{n^{+}-n^{-}}{n^{+}+n^{-}}$, where *n*^+ ^is the number of positive and *n*^- ^is the number of negative examples.

**Figure 1:**
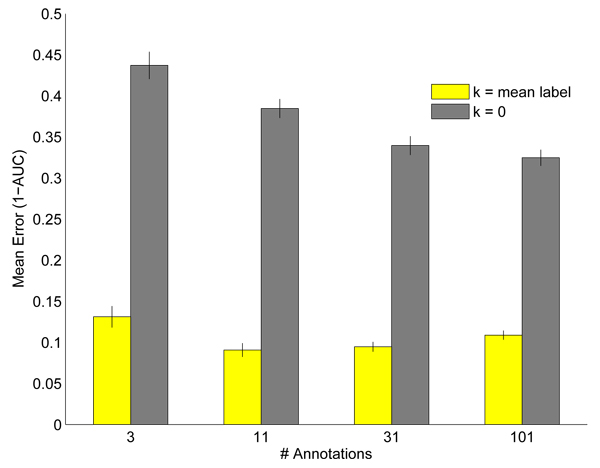
Effect of label bias on ROC scores. Bars show the prediction error measured using 1 - area under the receiver operating characteristic (ROC) curve (1 - AUC) of GeneMANIA where the label bias of unlabeled genes is set to zero or average label (that is, k = 0 or k = mean label). The experiments were run on 400 Gene Ontology (GO) functional classes using 15 yeast association networks that we created from various genomics and proteomics data sources (see Materials and methods). The functional classes are grouped by specificity (defined by number of annotated genes: 3 to 10, 11 to 30, 31 to 100, 101 to 300). Error bars depict the standard error on 100 different predictions in each evaluation category.

When the number of positively and negatively labeled genes is equal, the GeneMANIA label propagation algorithm is identical to that of Zhou and coworkers [22] (hereafter ZBLWS), and similar to the original Gaussian fields label propagation algorithm [23], since they both assign an initial label bias of 0 to the unlabeled nodes. However, in gene function prediction problems, the number of positives is almost always only a very small proportion of the total number of genes and our method of setting these label biases makes a dramatic improvement in prediction accuracy in these cases (Figure 1).

### Efficient implementation of GeneMANIA label propagation for large genomes

In GeneMANIA, label propagation on the composite association network is the most time-consuming step. Here we describe how we implemented this algorithm in order make it more efficient.

The discriminant values resulting from GeneMANIA label propagation can be calculated by solving a system of linear equations, ***y ***= *A****f***, for ***f***, the vector of discriminant values, given *A*, the coefficient matrix (whose derivation from the association network weights is described in Materials and methods) and ***y***, the vector of node label biases. While in principle this system can be easily solved by multiplying the inverse of the coefficient matrix by ***y***, we employ a conjugate gradient (CG) method to solve this system that makes efficient use of CPU processing time and computer memory resources, especially for large genomes.

To show why the CG method is well-suited for this problem, we will briefly describe the algorithm. The CG method iteratively improves an estimate, ***f***^***t***^, of a solution to the linear system as follows: at each iteration *t*, the current estimate, ***f***^***t***^, is multiplied by the matrix *A*. If the result of this matrix multiplication ***y***^***t ***^= *A****f***^***t ***^is equal to ***y ***then ***f***^***t ***^is a correct solution. On the other hand, if ***y***^***t ***^does not equal ***y ***then the CG method calculates a new estimate, ***f***^***t*+1**^, based on the difference between ***y***^***t ***^and ***y***. Calculating ***f***^***t*+1 **^requires performing another matrix multiplication between *A *and a vector with the same number of elements as ***f***^***t***^. The new estimate, ***f***^***t*+1**^, is guaranteed to be more accurate than ***f***^***t ***^[29] and, starting from a random estimate of ***f***, the CG method is guaranteed to converge to a correct solution after *n *iterations, where *n *is the number of nodes in the network. In practice, however, the CG method can converge in many fewer iterations.

**Figure 2:**
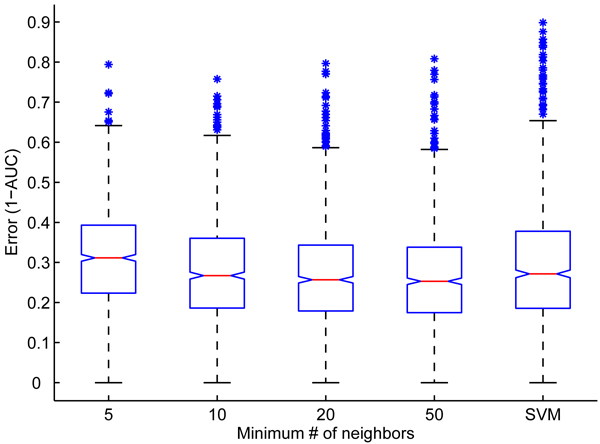
Effect of network sparsification on ROC scores. For various sparsity levels of GeneMANIA and for the support vector machine (SVM), boxplot shows the following features of the distribution of the prediction errors as measured with 1 - area under the receiver operating characteristic (ROC) curve (1 - AUC): the median (red line), 25% and 75% percentile (blue box), and outliers of prediction errors more than 1.5 times the interquartile range away from the median (blue stars). The evaluations are based on 3-fold cross-validation on 992 GO categories with the Zhang and coworkers [12] mouse tissue expression data as input. The GeneMANIA experiments were run by creating an association network from the mouse tissue expression data where the number of neighbors for each gene is restricted to N. For example, when the number of neighbors = 5, each gene is associated with only five other genes. The settings for the SVM experiments are as described in [12].

By reducing the number, *m*, of non-zero elements in *A*, we can reduce the runtime of the CG method for label propagation. The runtime of each CG iteration is proportional to *m*, which is equal to the number of edges plus the number of nodes in the functional association network that *A *represents. Many types of functional association networks are sparsely connected, and those that are not, such as networks derived from molecular profiling data, can be made sparse with little resulting impact on the performance of GeneMANIA (Figure 2). In contrast to the CG method, the run times of standard methods for solving linear systems, like Gaussian elimination, depend on the number of potential connections, which can be orders of magnitude larger than the number of actual ones.

**Figure 3:**
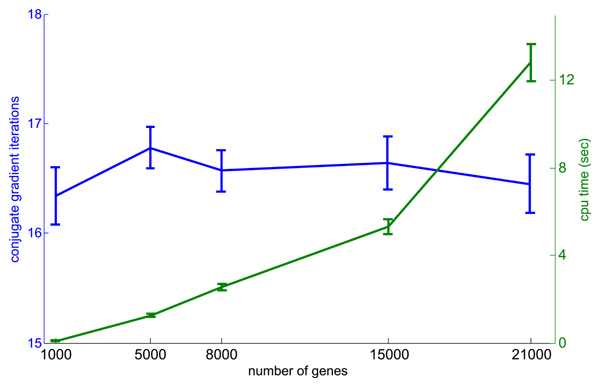
The number of CG iterations and computation time of the GeneMANIA algorithm as a function of number of genes. Left axis: the number of conjugate gradient (CG) iterations until convergence as a function of number of genes in the association networks. Right axis: computation time of GeneMANIA as a function of number of genes in the association networks. Experiments were run using ten association networks from the MouseFunc I benchmark data. The final point on the plot used the full mouse gene complement (for which data are available), and the other gene numbers were derived by taking random subsets of the full gene complement. Distribution is over 100 randomly selected Gene Ontology (GO) categories. The maximum number of CG iterations observed in any test was 20 and the maximum computation time was 15 seconds. The quadratic dependence of computation time on gene number is due to the quadratic growth in number of non-zero association links in the networks as a function of gene number (data not shown).

Using the CG method for label propagation leads to further time reductions because it is possible to get very close to the exact solution with only a fraction of the full number of required iterations. Indeed, we found that for networks with sizes ranging from 1,000 nodes to 20,000 nodes, on average less than 20 CG iterations were needed to derive a solution that is within the computer round-off error of the exact solution (Figure 3). Because there does not appear to be any relationship between network size and number of CG iterations required to reach a solution in our application, in practice, the running time of our label propagation algorithm depends only upon *m*, in contrast to other methods for solving linear systems whose runtime in the worst case would depend on the product of *m *and *n*.

In summary, to make our algorithm efficient for large genomes, we use a conjugate gradient-based procedure to solve for the discriminant values and we sparsify functional association networks so that only the most informative associations are retained. These two changes speed up the label propagation algorithm by orders of magnitude for large genomes.

### GeneMANIA network integration

Like two recent methods for combining multiple functional association networks [9,17], GeneMANIA builds a composite functional association network by taking a weighted average of the individual functional association networks. However, unlike these two methods, GeneMANIA optimizes the network weights and calculates the discriminant values separately. The advantage of this approach is that unlike the algorithm described in Tsuda and coworkers [17] (hereafter TSS), which also uses Gaussian field label propagation, GeneMANIA needs to run the computationally intensive label propagation only once, whereas TSS runs it multiple times on different networks while computing the network weights. For example, on our five network yeast benchmark (described in Materials and methods) across 400 GO functional categories, the TSS runs label propagation, on average, 68.8 times per category.

Our approach to computing network weights was inspired by recent work showing that when all available functional association networks are relevant for predicting gene function, a composite network generated by weighting each data source equally supports predictions as accurate as those derived using a composite network generated by an optimal choice of weights [17,30], but when some of the association networks are irrelevant, the prediction performance of equal weighting scheme is degraded [30]. These observations suggest that heuristics network-weighting methods that can identify and down-weight irrelevant networks may be as accurate as optimal network weightings. As we show later, it is also important to be able to identify and down-weight redundant functional association networks.

The regularized linear regression algorithm that GeneMANIA uses is, by design, robust to the inclusion of irrelevant and redundant networks. This property is especially important when the data sources cannot be carefully controlled - for example, in a webserver that automatically downloads new data from web repositories or allows users to contribute their own data.

To set the network weights, we use ridge regression [31], which corresponds to finding a vector of network weights, ***α ***= [*α*_1_,..., *α*_*d*_]^*t*^, that minimizes the cost function: (***t ***- Ω***α***)^*t*^(***t ***- Ω***α***) + (***α ***- ***α***)^*t*^*S*(***α ***- ***α***). Here *α*_*i *_is the weight of the i^th ^network, ***t ***is a vector derived from the initial label of the labeled nodes, Ω is a matrix with columns corresponding to individual association networks, ***α ***is the mean prior weight vector, and ***S ***is a diagonal precision matrix. When all the diagonal entries of ***S ***are set to zero, we say that the cost function is unregularized and solving for *α *becomes equivalent to unregularized linear regression. For versions of the GeneMANIA algorithm designed to be deployed on a webserver, we have limited prior information about the networks and we set the mean prior weight of all networks to be equal. We call this the 'equal-weight' prior. However, when predicting membership in a GO functional category, we can improve prediction accuracies if we set the mean prior weight of each network to be the average of all the weight vectors calculated using unregularized linear regression on a large number of functional classes in the same branch of GO. We call this the 'branch-specific' weight prior (see Materials and methods for details).

### Gene function prediction in mouse

To evaluate GeneMANIA, we performed function prediction using the MouseFunc I benchmark data. To do so, we constructed ten association networks from the ten datasets (see Materials and methods for details) and used the GeneMANIA network integration and label propagation algorithm to predict gene function for each function class separately. When constructing individual association networks, we set the sparsity level to S = 50, that is, we kept only the top 50 association weights (links) for each gene and set the rest to zero. Setting S = 100 results in better performance on most specific GO biological process (BP) classes while it has minimal effect in other evaluation categories (supplementary Figure 2 in Additional data file 1).

We follow the same procedure as [15] and compare prediction accuracies using average area under the receiver operating characteristic (ROC) curve (AUC) in twelve MouseFunc I evaluation categories. Briefly, the evaluation classes are created by grouping GO categories corresponding to all pairwise combinations of the three GO branches (BP, cellular component [CC], and molecular function [MF]) and four specificity levels, that is, number of annotations ([3 to 10], [11 to 30], [31 to 100], and [101 to 300]). We report prediction performance on both the 'test' and the 'novel' benchmarks (see [15] for details).

We compare GeneMANIA's predictions in the first and second rounds of MouseFunc I (GeneMANIA^Entry-1 ^and GeneMANIA^Entry-2^, respectively) and the version of GeneMANIA designed to be implemented on a webserver (GeneMANIA^WS^) against the best performing methods in MouseFunc I. For GeneMANIA^WS^, we assume no knowledge about the source of the gene list whereas in MouseFunc I the competing methods were provided with the name of GO category from which each gene list was derived. Below we discuss the outcome of our evaluation on the 'test' benchmark and then on the 'novel' benchmark.

**Figure 4:**
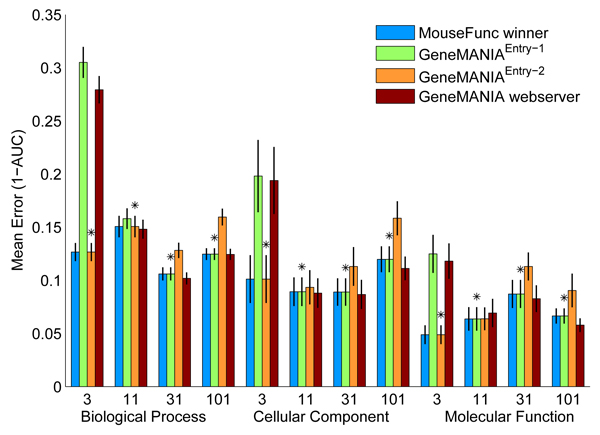
Prediction performance of GeneMANIA on the MouseFunc I test benchmark. Prediction performance of the first and second submissions to MouseFunc I (GeneMANIA^Entry-1 ^and GeneMANIA^Entry-2^, respectively) as well as the version of the GeneMANIA algorithm we have implemented on the GeneMANIA webserver (GeneMANIA^WS^) and the best achieved performance on the MouseFunc I test benchmark. Prediction performance is indicated by mean 1 - area under the receiver operating characteristic curve (1 - AUC) in the class, error bars show one standard error of the mean. Stars mark the evaluation classes in which a GeneMANIA entry achieved lowest error on the test benchmark.

Figure 4 shows the prediction performance of three versions of GeneMANIA as well as the best performance that was achieved on the test benchmark; GeneMANIA^Entry-1 ^achieved the highest prediction performance on all evaluation classes containing GO categories with more than ten annotated genes, and GeneMANIA^Entry-2 ^achieved the highest prediction performance on the categories with ten or fewer annotated genes. We measure performance by using one minus the AUC (1 - AUC), so lower values indicate better performance. GeneMANIA^Entry-2 ^had significantly lower median 1 - AUC than all other methods in predicting BP and MF categories with 3 to 10 annotations (*p *< 0.05, Wilcoxon-Mann-Whitney) while GeneMANIA^Entry1 ^and GeneMANIA^WS ^had significantly lower median 1 - AUC compared with GeneMANIA^Entry-2 ^in predicting BP categories with 31 to 100 and 101 to 300 annotations, CC categories with 101 to 300 annotations, and MF categories with 31 to 100 annotations.

The reduced performance of GeneMANIA^Entry-1 ^in the categories with the fewest annotations is likely due to overfitting, because we used unregularized linear regression to set the network weights. In GeneMANIA^Entry-2^, we switched to ridge regression with the branch-specific weight prior, which is less prone to overfitting. Note also that GeneMANIA^WS^, which uses an equal-weight prior, also has improved performance on the smaller GO categories.

**Figure 5:**
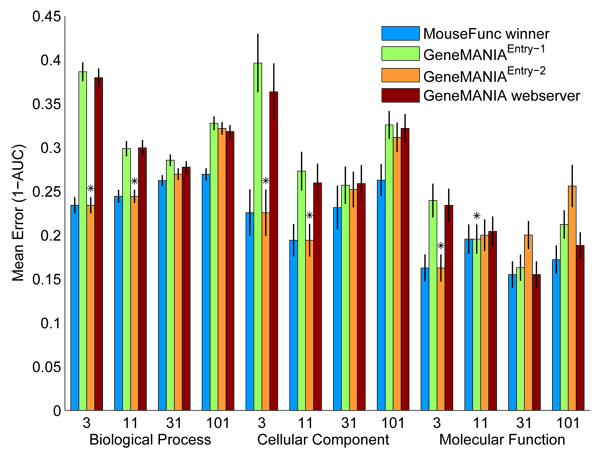
Prediction performance of GeneMANIA on the MouseFunc I novel benchmark. Prediction performance of the first and second submissions to MouseFunc I (GeneMANIA^Entry-1 ^and GeneMANIA^Entry-2^, respectively) as well as the version of the GeneMANIA algorithm we have implemented on the GeneMANIA webserver (GeneMANIA^WS^) and the best achieved performance on the MouseFunc I novel benchmark. Stars mark the evaluation categories in which GeneMANIA entries had the best achieved performance on the test benchmark. Bars show mean error (measured as 1 - area under the receiver operating characteristic curve [1 - AUC]), error bars indicate one standard error.

We suspect that one reason for the drop in prediction performance of GeneMANIA^Entry-2 ^compared with GeneMANIA^Entry-1 ^on the larger GO categories in the test benchmark is because of our definition of negative examples. In our first entry, we defined as negative examples all genes with any GO annotation but not one in the category being predicted. In our second entry, we refined this definition so that the negative examples for a given category were only those annotated to a sibling category, that is, one that shared a parent in the GO hierarchy. Although choosing the genes annotated to sibling categories of interest as negatives improved prediction performance on novel tasks (Figure 5), it degraded the prediction performance on the test set. This may due to the reduction in the number of negative examples. One way to alleviate this effect is to define a range of label biases between [-1, 0] for genes that have GO annotations but are not annotated to the function of interest.

On the novel benchmark, in addition to the ten association networks, eight month out-of-date annotations for test genes were also made available. Annotations in other categories of gene function can be informative for predicting new annotations in a given category (for example [32]); in our second entry to MouseFunc I, we investigated two ways in which old annotations may be helpful in predicting new annotations on the novel benchmark. First, we reasoned that genes previously annotated in parent categories but not sibling categories should be considered as potential new positive examples and as such, as described earlier, we used only genes annotated to sibling categories as negatives. Second, often annotations in one branch of the GO imply annotations in a different branch. For example, transcription factors (MF annotation) are located in the nucleus at least some of the time (CC annotation) and a number of transcription factors are involved in cell cycle regulation (BP category). To include this information, we constructed association networks from each branch of GO (see Materials and methods).

Figure 5 shows the prediction accuracies of three versions of GeneMANIA as well as the best accuracy obtained for each evaluation set on the 'novel' MouseFunc I benchmark. For the evaluation classes containing the GO categories with 3 to 10 or 11 to 30 annotations, GeneMANIA entries still had the lowest error on the MouseFunc I benchmark, with GeneMANIA^Entry-2 ^having significantly smaller median error in five of the six 'small-sized' evaluation categories (*p *< 0.05, Wilcoxon-Mann-Whitney). GeneMANIA did less well in the larger GO categories, possibly suggesting that we are not making full use of the older annotations. In contrast to the test benchmark, here, GeneMANIA^Entry-2 ^improved performance can be mostly attributed to the choice of negative examples (data not shown), although the inclusion of the GO networks also slightly improves the prediction performance (data not shown).

In summary, we observed that employing regularization when using linear regression to combine multiple networks results in a drastic improvement in prediction accuracies in most specific functional classes. This is because with only a few positive examples, identifying relevant networks is a challenging task and prone to over-fitting. One way of alleviating this effect is to estimate the relevancy of each network based on its average weight on a large number of similar prediction tasks (for example, prediction of functional classes in the same branch of GO). Second, we have demonstrated that in the binary classification of genes according to GO classes, the genes that are used as negative examples have a large impact on the prediction outcome with label propagation.

### Performance of GeneMANIA on the yeast benchmark

Inspired by the performance of GeneMANIA on the MouseFunc I benchmark, we compare GeneMANIA's performance to that of the TSS algorithm and bioPIXIE [14] on yeast data. To compare GeneMANIA with the TSS algorithm, we used five yeast functional association networks (from [9]) and 400 yeast GO functional classes that we derived from a 2006 version of GO annotations (see Materials and methods for details). We compare GeneMANIA with the TSS algorithm, which had previously been shown to be as accurate as an SVM with optimized network weights while requiring orders of magnitude less computation time [17], enabling us to run much more extensive comparisons. We compared GeneMANIA with the bioPIXIE network because it is currently deployed on a popular website that provides gene function predictions. For bioPIXIE, we evaluate both the network and the probabilistic graph search algorithm published with the bioPIXIE network in comparison with GeneMANIA. Note that the bioPIXIE network was built using different data sources, which are not included in the five yeast network benchmark, so the reported performance is with regard to the specific published network, not the Bayesian network algorithm used to derive the network. In addition, to illustrate the attainable accuracies with additional datasets, we performed function prediction with GeneMANIA using a 15 yeast benchmark data set (GeneMANIA^15^) that we derived from recent genomics and proteomics data sources (see Materials and methods). However, we were unable to use these networks as input to the TSS algorithm due to the memory requirements of the algorithm.

**Figure 6:**
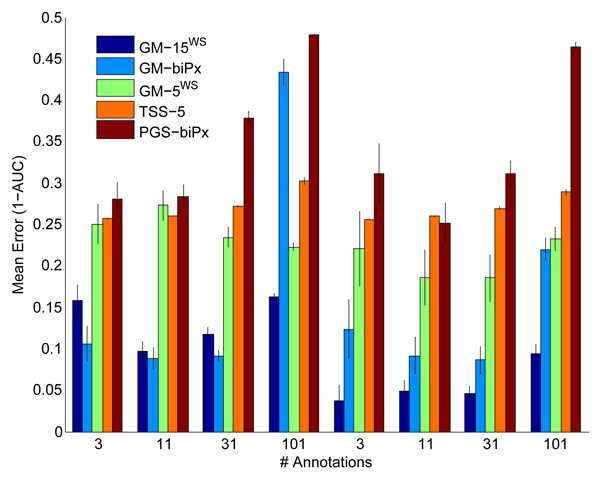
Prediction performance of GeneMANIA on the extended yeast benchmark. Prediction performance of GeneMANIA with five yeast networks with equal weight prior (GM-5^WS^), 15 yeast networks with equal weight prior (GM-15^WS^), GeneMANIA with the bioPIXIE network (GM-biPx), and the TSS algorithm with five yeast networks (TSS-5). Bars show mean error (measured as 1 - area under the receiver operating characteristic curve [1 - AUC]) on 12 evaluation classes based on ontologies (biological process [BP] and cellular component [CC]) and specificity levels of 3 to 10, 11 to 30, 31 to 100, and 101 to 300 annotations. Error bars indicate the standard error in the mean.

Figure 6 shows the prediction performance on the yeast benchmark on BP and CC evaluation classes (see supplementary Figure 4 in Additional data file 1 for results on MF functional classes) for the TSS algorithm, bioPIXIE and GeneMANIA on a variety of input collections of functional association networks. The comparative performance of GeneMANIA^15 ^(GM-15^WS^) to GeneMANIA label propagation applied to the bioPIXIE network (GM-biPx) and to the bioPIXIE probabilistic graph search (PGS) algorithm applied on the same network (PGS-biPx) underlines the value of the GeneMANIA algorithm for a webserver. Given the bioPIXIE network as input, GeneMANIA has a significantly (*p *< 0.05, Wilcoxon-Mann-Whitney) lower median 1 - AUC than PGS-biPx in all evaluation classes, showing that using simple heuristics to assign discriminant values leads to a considerable loss in accuracy. The value of being able to tailor the composite functional association network to the input seed list is shown by the comparative performance of GM-biPx and GM-15; while GM-biPx performs well compared with GM-15 in predicting the smaller GO-BP categories, for which the bioPIXIE network was optimized, GM-biPx has much higher error, and significantly lower performance (*p *< 0.05, Wilcoxon-Mann-Whitney) for the largest GO-BP categories and GO categories from other branches of the hierarchy that the bioPIXIE network was not designed to predict. In our tests, GM-biPx outperforms GM-15 on the smaller GO-BP categories; however, the performance of GM-biPx is likely overestimated because the test sets used for evaluating GM-biPx are not independent from the training sets used to build the bioPIXIE network since we were not able to recover the cross-validation folds used in [14].

We were not able to compare GeneMANIA with the TSS algorithm on the 15 yeast network benchmark; the TSS algorithm (as obtained from [33]) run on the 15 yeast networks resulted in an out of memory error when solving the system of linear equations when running MATLAB (version 7.4) on a quad-core MAC desktop with Intel Xeon 2.66 GHz processors and 4 GB of memory. However, when comparing GeneMANIA on the five yeast networks from [17] (TSS-5 versus GM-5), we see that GeneMANIA performs as well as or better than TSS; among the evaluation categories shown in Figure 6, GM-5 has a significant lower median 1 - AUC than TSS-5 on BP and CC categories with 31 to 100 and 101 to 300 annotations (*p *< 0.05, Wilcoxon-Mann-Whitney).

### Running time and prediction accuracy

**Figure 7:**
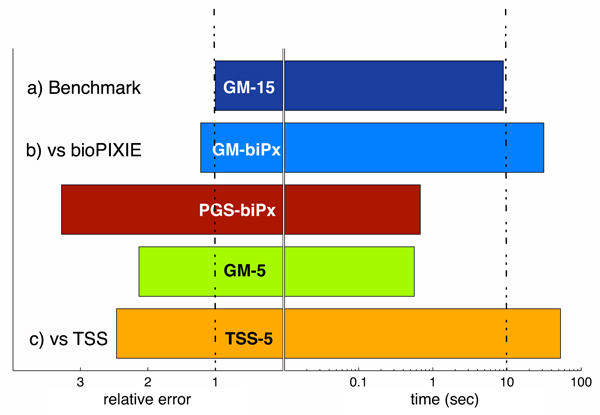
Computation time and prediction accuracy. Bars to the left of the solid vertical lines show fold increase in error relative to the mean error (as measured by 1 - area under the receiver operating characteristic curve [1 - AUC]) for the evaluation classes defined in Figure 6 of GeneMANIA with 15 yeast networks using the branch-specific weight priors in Figure 6. Bars to the right of the solid vertical lines show mean CPU time required to run each algorithm. The performance of GeneMANIA (GM), TSS, and bioPIXIE (biPx) are directly compared on the same input. The bars marked as GM-biPx and PGS-biPx depict the prediction performance of GeneMANIA label propagation and bioPIXIE probabilistic graph based search algorithm, respectively, using the bioPIXIE network as input. TSS and GeneMANIA are compared using the five yeast network benchmark.

Figure 7 demonstrates the trade-off between computation time and mean error of GeneMANIA, the TSS algorithm, and the bioPIXIE graph search algorithm on the yeast benchmark. GeneMANIA with 15 networks and branch-specific weight prior achieves the lowest error in yeast and we use it as a benchmark for comparing the error of other methods. While PGS-biPx was faster than GM-biPx, its median error (1 - AUC) was three times higher. We also note that the TSS-5 algorithm was both slower and, on average, less accurate than GM-5. The primary cause of this slow down is that the TSS algorithm runs label propagation more than 50 times more than GeneMANIA in order to optimize the network weights.

### The effect of redundancy and random networks on equal weighting

**Figure 8:**
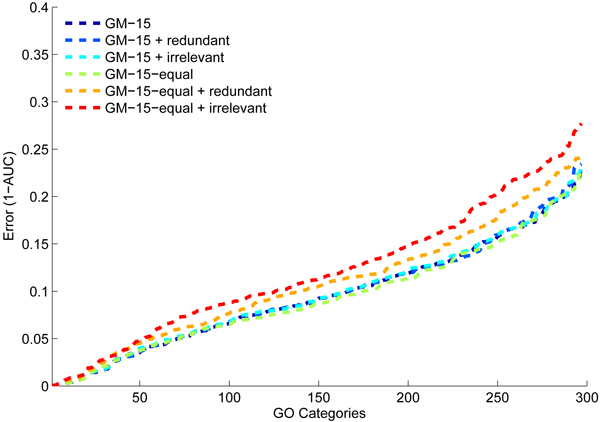
Prediction performance of GeneMANIA in the presence of irrelevant and redundant networks. Cumulative distribution of 1 - area under the receiver operating characteristic curve (1 - AUC) scores on 300 yeast Gene Ontology (GO) categories using GeneMANIA optimized weights and equal weights in the presence of redundant and irrelevant networks.

To ensure that the GeneMANIA network integration scheme is robust to irrelevant and redundant networks, we evaluated GeneMANIA in the presence of redundant and noisy networks. To do so, we constructed 20 redundant yeast networks by adding a slight amount of noise to the PfamA network (Gaussian noise with mean zero and standard deviation of 0.025). We also constructed two irrelevant networks by assigning association weights between 0 and 1, drawn from a uniform distribution, to a random set of 0.01% of the association weights and setting the rest of the associations to zero. We conducted function prediction with the GeneMANIA network integration scheme and weight scheme in which all the networks are assigned an equal weight. Figure 8 shows the results of function prediction with these two strategies on 300 yeast GO categories with 11 to 300 annotations. In the presence of irrelevant and redundant networks, the performance of the equal weight scheme is drastically degraded. The multiple network integration scheme of GeneMANIA detects these redundant and irrelevant networks and, as a result, GeneMANIA's performance is not affected.

## Discussion and conclusion

We have shown that GeneMANIA is as accurate as, or more so than, leading gene function prediction algorithms on yeast and mouse despite requiring, in some cases, orders of magnitude less computation time. We achieve the highest accuracy using a version of our algorithm that requires between 10 and 15 seconds computation time on a modern desktop computer with slightly optimized MATLAB code. With more careful optimization, we expect that GeneMANIA will be even faster. Consequently, we have demonstrated that it is possible to design a gene function prediction algorithm that performs on-demand function prediction with most up-to-date annotation list and data sources while achieving the same or better accuracy as the leading algorithms.

Our algorithm automatically combines multiple input data sources fast enough to be employed on a web server. With the availability of such an algorithm, it is no longer necessary to maintain static databases of gene function predictions that can quickly become out of date and are restricted to predicting function in pre-defined categories. As proof of this principle, we have generated a website [34] where users can define their own function categories by providing a gene list, and receive predictions in seconds.

Additionally, we have demonstrated that sparsifying data sources can result in increased prediction accuracy as well as decreased computation time. This suggests that, in the context of gene function prediction, many of the derived associations may be uninformative and only a small selection of a gene's top neighbors are sufficient for accurate function determination.

Here we have not investigated the possibility of using a gene's prior annotations to predict new ones; this type of information can be useful [32], although it can easily be incorporated by using it to generate a functional association network, as we did in our MouseFunc I entry. We have also noticed that, in some cases, a network representation is not the most efficient encoding of input data; for example, our yeast gene localization dataset has a sparse feature-based representation and a dense network-based representation. This can be addressed using techniques proposed in [35], which allows the propagation of label information on networks where each node is also associated with a feature vector.

## Materials and methods

### Software

The GeneMANIA prototype webserver is available at [34].

### Yeast benchmark datasets

To perform function prediction in yeast with the GeneMANIA algorithm, we used five yeast functional association networks (used in [9] and [17] and available from [33]), constructed an extended yeast benchmark consisting of 15 association networks and 400 GO functional classes, and used the bioPIXIE network (obtained from [36]).

We constructed 15 yeast association networks from various genomics data sources (supplementary Table 1 in Additional data file 2). In addition, we also downloaded the GO association file from the *Saccharomyces *Genome Database on (1 June 2006) and obtained a set of 400 functional classes that we grouped according to their specificity level and GO branch. In particular, we randomly selected 100 functional classes from each specificity level (3 to 10, 11 to 30, 31 to 100, and 101 to 300) and organized them based on their GO branch. In addition, the 5 yeast network benchmark consists of 13 Munich Information Center for Protein Sequences (MIPS) functional classes. We have included the prediction performance on these functional classes in supplementary Figures 3 and 5 in Additional data file 1.

In addition to the above, we investigated the prediction performance of GeneMANIA using the bioPIXIE [14] network (we refer to this method as GeneMANAI^bioPIXIE^). bioPIXIE is a composite association network that has been constructed from 925 referenced data sources. It contains 15,551,081 functional associations between 7,034 yeast genes.

### Zhang and coworkers mouse benchmark data

We obtained the mouse tissue expression benchmark data from [37]. For comparison of GeneMANIA with the benchmark data from [12], following their experimental framework, we considered 9,499 genes and 992 GO categories.

### MouseFunc I benchmark data

We constructed ten association networks from the MouseFunc I benchmark data (as detailed below). We used minor variations of GeneMANIA for our evaluations: GeneMANIA^Entry-1^, GeneMANIA^Entry-1^, and GeneMANIA^WS^.

In GeneMANIA^Entry-1 ^we used unregularized linear regression to combine association networks, and we used all genes with any GO annotations that are not annotated to the functional class of interest as negative examples.

In GeneMANIA^Entry-2 ^we used ridge regression to combine association networks, and we set the prior weight vector *α*_*h *_for network *h *to the average weight that network *h *obtained when using ordinary linear regression in all predictions from the same GO branch. We define negative examples for each class of interest *g *as those genes that have annotations to any class that shares a parent with *g*. In addition, we included three GO networks that were constructed from three GO ontologies.

GeneMANIA^WS ^is the same as GeneMANIA^Entry-1 ^except that we used ridge regression, setting all of the elements of the mean prior weight vector to *1*/*d *where *d *is the number of interaction networks.

### Building functional association networks

When constructing association networks, we distinguish two types of data: binary and continuous valued. In binary datasets, for a given feature, all zeros were replaced with *log*(1 - *β*) and ones were replaced with, -*log*(*β*), where *β *is the proportion of examples for which the given feature has a value of 1. This allows for the emphasis of similarities between genes that share 'uncommon' features. Subsequently, for both types of data, we constructed similarity matrices using the Pearson correlation coefficient to measure pair-wise similarities. To keep our association networks sparse, for each gene we set all of its links (association weights), except for its top 50 links, to zero.

### Mouse GO networks

For predicting gene function on the MouseFunc I benchmark, in addition to the ten association networks that we constructed from the genomics and proteomics data, we constructed three GO networks. We represented a gene's annotations to a given branch of GO as a binary vector whose *g*^*th *^element is set to '1' if the gene was annotated in GO category *g*, or any of the descendants of *g *in the GO hierarchy, and is set to '0' otherwise. We constructed one GO network for each branch of the hierarchy. However, when predicting GO category *g *in ontology BP (CC, or MF), we would construct the BP GO network by not including any annotations in *g *or its descendents.

### Network normalization

We normalize all our functional association networks and the composite process-specific network by dividing each entry *W*_*ij*_, the association between node *i *and node *j*, by the square root of the product of the sum of the elements of row *i *and the sum of the elements in column *j *of the unnormalized network.

### Figure of merit for accuracy

We evaluated the performance of GeneMANIA using the AUC.

ROC is a graphical plot of true positive rate (sensitivity) as a function of false positive rate (1 - specificity) for a binary classifier system as its discrimination threshold is varied; a perfect classifier would yield an AUC of 1 and a random classifier yields an AUC of 0.5 [38]. 1 - AUC is a more appropriate measure because it corresponds to the expected portion of the negatives that are higher than a random positive - while a gain of 0.90 to 0.95 AUC seems negligible, it corresponds to a two-fold reduction in the expected portion of negatives scoring higher than a random positive (1 - AUC).

### GeneMANIA label propagation algorithm

Here we denote the vector of discriminant values by ***f***, the bias vector by ***y***, and the matrix derived from the association network by W. We can represent an association network over *n *genes by a symmetric matrix W whose non-zero entries indicate the associations in the network. In particular, the (*i*, *j*)^th ^element of W, *W*_*ij*_, is the association between genes *i *and *j*, with *W*_*ij *_= 0 indicating no edge between genes *i *and *j*. To ensure that all associations are non-negative, we set any negative associations to zero. For each binary classification task, we have *l *labeled genes and *u *unlabeled genes (*n *= l + *u*). We use these labels to specify a bias vector ***y***, where *y *∈ {+1, *k*, -1}, indicating that gene *i *is positive, unlabeled, or negative, respectively. In the GeneMANIA label propagation algorithm:

$$k=\frac{n^{+}-n^{-}}{n}$$

where *n*^+ ^and *n*^- ^are the numbers of positive and negative genes, respectively. The discriminant values are computed by solving the following objective function:

(1) $$f={\text{argmin}}_{f}\;\sum_{i}{\left(f_{i}-y_{i}\right)}^{2}+\sum_{i}\sum_{j}w_{ij}{\left(f_{i}-f_{j}\right)}^{2}$$

which ensures that the discriminant values of positive and negative genes remain close to their label bias (first term in the summation) and the discriminant values of genes that are associated (genes that have positive *w*_*ij*_) are not too different from each other (second term in the summation). Equation 1 can be written in matrix notation as:

f* = argmin_*f *_(***f ***- ***y***)^*t *^(***f ***- ***y***) + ***f***^*t *^*L****f***

where *L *= *D *- *W *is called the graph Laplacian matrix and *D = diag*(*d*_*i*_) (that is, D is a diagonal matrix with *D*_*ii *_= *d*_*i *_and *D*_*ij *_= 0 if *i *≠ *j*) and *d*_*i *_= Σ_*j*_*w*_*ij*_. Since the association matrix is symmetric, by definition, *L *is symmetric and semi-definite positive and equation 2 is a quadratic optimization problem with a global minimum. In fact, the solution to equation 2 can be obtained by solving a sparse linear system *y *= (***I ***- *L*)***f***.

### GeneMANIA network combination

Given *d *association networks encoded as matrices *W*_1_,.., *W*_*d*_, we form a composite network as:

$$W^{comb}=\sum_{h}\alpha_{h}W_{h}$$

where the vector of network weights, ***α ***= [*α*_1_, *α*_2_,..., *α*_*d*_], is customized to a particular prediction task by choosing it to maximize a form of kernel-target alignment [39] between the composite network and a 'target' network constructed from the class label vector ***y***.

Specifically, to obtain the weight vector ***α***, we solve the ridge regression problem:

***α ***= argmin_***α'***_(Ω***α' ***- ***t***)^*t*^(Ω***α' ***- ***t***) + (***α' ***- ***t***)^*t*^*S*(***α' ***- ***α***)

where each column Ω_*h *_of Ω constructed from the matrix *W*_*h *_by collecting its elements *W*_*ij *_for which either: one of the genes *i *or *j *is labeled positive and the other negative; or both are labeled positives. Each element of the vector ***t ***also corresponds to either a pair of negatively and positively labeled genes or two positively labeled genes. Elements of ***t ***corresponding to a positive and a negative are set to -*n*^+ ^× *n*^- ^and those corresponding to two positives are set to *n*^- ^× *n*^-^, where *n*^+ ^and *n*^- ^are the numbers of positively and negatively labeled genes, respectively. The same row ordering is used for all Ω_*h *_and ***t***, so that each row corresponds to a single *i*, *j *gene pair. When solving equation 3, we also include a bias term, *α*_0_, by adding a column of '1's, Ω_0_, to Ω and discard this bias term when constructing the composite association network. Intuitively, we are attempting to choose network weights such that pairs of positively labeled genes have high similarity, pairs containing a positive and negative gene have low similarity, and pairs of negatively labeled genes have no influence in determining the weights.

Given the prior mean vector ***α*** and precision matrix *S*, the solution to equation 3 is:

***α ***= (Ω^*t*^Ω + *S*)^-1^(Ω^*t*^***t ***+ *S****α***)

where Ω^*t *^indicates the transpose of Ω In the text, we describe two different methods to set ***α***, and we specify *S* by setting all its elements to zero, except for its diagonal elements, which we specify by setting *S*_*hh *_= trace(*W*_*h*_^*t*^*W*_*h*_). If we are solving the unregularized version of this problem, we simply set all of the elements of *S* to zero.

To avoid negative network weights, if after solving for ***α***, *α*_*h *_< 0 for any *h *> *0*, then we set *α*_*h *_= 0, remove Ω_*h *_from Ω, and redo the ridge regression. If through this procedure, *α*_*h *_= 0 for all *h *> 0, we set *α*_*h *_= 1/*d *for all *h *> 0.

The time complexity of this algorithm scales at worst quadratically in *n*. Computing equation 3 requires O(*n *× *n*^+ ^× *d*) time to calculate Ω^*t*^Ω and the matrix inversion can be done in O(*d*^3^) time.

### The TSS algorithm

We downloaded the TSS algorithm from [33]. There are three parameters to be set: *C *is the constraint that enforces an upper-bound on the sum of the network weights (that is, $\sum_{k}\alpha_{k}\leq C$), *C*_0 _enforces and upper-bound on individual network weights (that is, 0 ≤ *α*_*k *_≤ *C*_0_), and *const *is a parameter to adjust for the class imbalances. In the code provided by [33], the parameters *C *and *C*_0 _have been previously optimized for function prediction in the 13 MIPS categories for the five yeast network input. For the 400 GO category yeast benchmark, we set these parameters to their default values (that is, *C*_0 _= 0.4).

### bioPIXIE probabilistic graph search algorithm

Given a seed list of positive genes, the probabilistic graph search algorithm adds two sets of genes to it. The first set contains the *n*_1 _genes with the highest total association weight to the seed list and the second set contains the *n*_2 _genes with highest total association weights to the first set. In [14], the authors set *n*_1 _to be between 10 and 20 and *n*_2 _to be 40 - *n*_1_. In our implementation of [14], we set both *n*_1 _and *n*_2 _to 20. We chose these two parameters empirically, by comparing several settings in the suggested range. Our reported results are robust to slight variation of these two parameters.

## Abbreviations

AUC, area under the ROC curve; BP, biological process; CC, cellular component; CG, conjugate gradient; GO, Gene Ontology; MF, molecular function; MIPS, Munich Information Center for Protein Sequences; PGS, probabilistic graph search; ROC, receiver operating characteristic; SVM, support vector machine; TSS, Tsuda and coworkers [17] algorithm; ZBLWS, Zhou and coworkers [22] algorithm.

## Competing interests

The authors declare that they have no competing interests.

## Authors' contributions

QM conceived of the project. QM, DR, and SM designed the GeneMANIA algorithm. SM and DWF implemented the GeneMANIA algorithm. DWF implemented the GeneMANIA webserver. SM collected the data, designed the association networks, and performed the experiments. CG contributed code to create association networks. SM and QM wrote the manuscript. QM supervised the project and provided feedback.

## Additional data files

The following additional data are available with the online version of this paper. Additional data file 1 includes supplementary Figures 1 to 5. Additional data file 2 includes supplementary Table 1.

## Supplementary Material

Additional data file 1 Supplementary Figures 1 to 5. Click here for file

Additional data file 2 Supplementary Table 1. Click here for file
