# Supplementary material for: GeneMANIA: a real-time multiple association network integration algorithm for predicting gene function
Source: Genome Biol. 2008 Jun 27;9(Suppl 1):S4. doi: 10.1186/gb-2008-9-s1-s4 (PMC2447538; doi:10.1186/gb-2008-9-s1-s4)
Supplement: Additional data file 1 — Supplementary Figures 1 to 5. [file gb-2008-9-s1-s4-S1.pdf]

| | | |
|---|---|---|
| 1 | Co-expression (cell cycle) | Spellman et al. Mol Biol Cell. (1998). 9 (12):3273-97. |
| 2 | Co-expression (Deletion mutants) | Hughes et al. Cell (2000). 102(1):109-26. |
| 3 | Co-expression (Environmental stress) | Gasch et al. Mol Biol Cell (2000). 11(12):4241-57. |
| 4 | Co-regulated (ChIP-chip rich media) | Lee et al. Science (2002). 298(5594): 799-804. |
| 5 | Co-regulated (ChIP-chip environmental stress) | Harbison et al. Nature (2004). 431(7004):99-104. |
| 6 | Haploinsufficiency profiling | Giaever et al. Proc Natl Acad Sci USA (2004). 101(3):793-8. |
| 7 | Shared protein domains (PfamA) | Downloaded September, 2006 |
| 8 | Shared protein domains (PfamB) | Downloaded September, 2006 |
| 9 | Co-expression (Essential deletion mutants) | Chua et al. Proc Natl Acad Sci USA (2006). 103(32):12045-50. |
| 10 | Co-expression (Overexpression mutants) | Chua et al. Proc Natl Acad Sci USA (2006). 103(32):12045-50. |
| 11 | Co-expression (Pheromone response) | Roberts et al. Science (2000). 287(5454):873-80. |
| 12 | Co-complexed (PPI) | Gavin et al. Nature (2006). 440 (7084): 631-6. |
| 13 | Co-complexed (PPI) | Krogan et al. Nature (2006). 440(7084): 637-43. |
| 14 | Co-expression (Yeast outcrosses) | Yvert et al. Nat Genetics (2003). 35(1): 57-64. |
| 15 | Co-localization | Huh et al. Nature (2003). 425(6959):686-91. |
