# Supplementary material for: GeneMANIA: a real-time multiple association network integration algorithm for predicting gene function
Source: Genome Biol. 2008 Jun 27;9(Suppl 1):S4. doi: 10.1186/gb-2008-9-s1-s4 (PMC2447538; doi:10.1186/gb-2008-9-s1-s4)
Supplement: Additional data file 2 — Supplementary Table 1. [file gb-2008-9-s1-s4-S2.pdf]

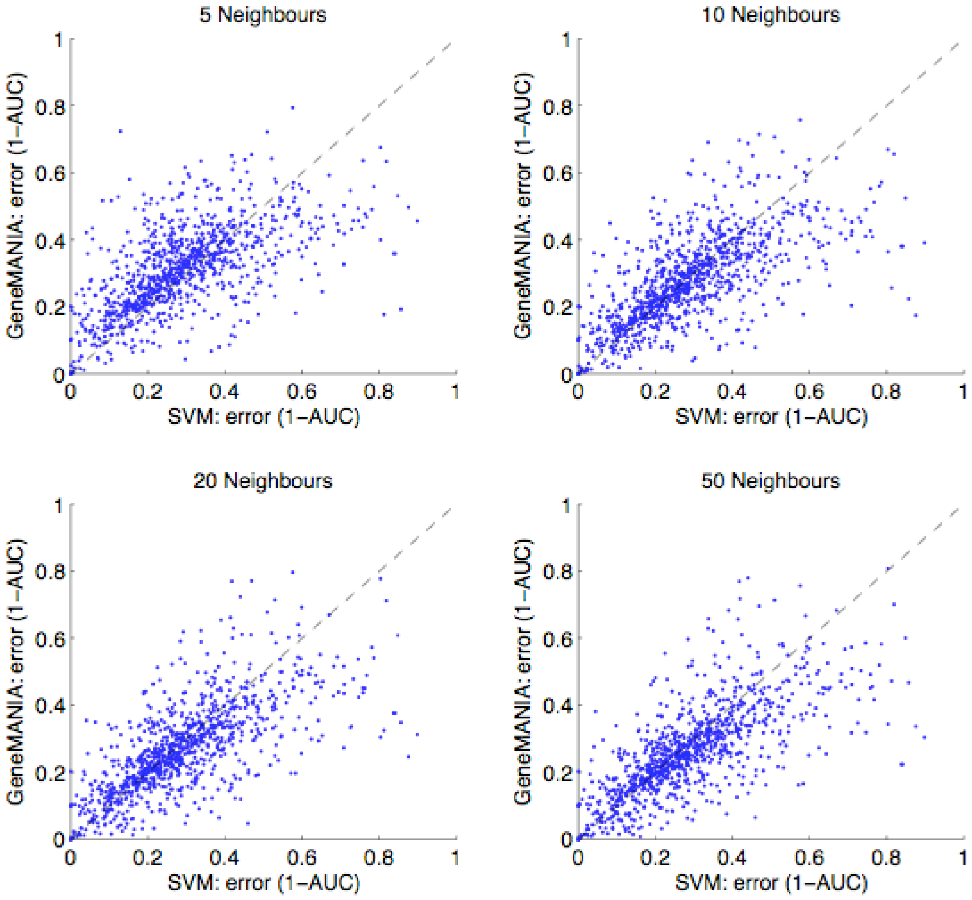

Figure S1. Effect of Network Sparsification on prediction error. Scatter plots show error (1-AUC) on predicting 992 GO categories using GeneMANIA and SVM. For predicting gene function with GeneMANIA, we varied the number of neighbours in the input network (mouse gene expression data from Zhang et al. [12]). For predicting gene function with SVM, we used the same setting as in [12]. When number of neighbours is greater than 10, GeneMANIA performs as well or better than SVM.

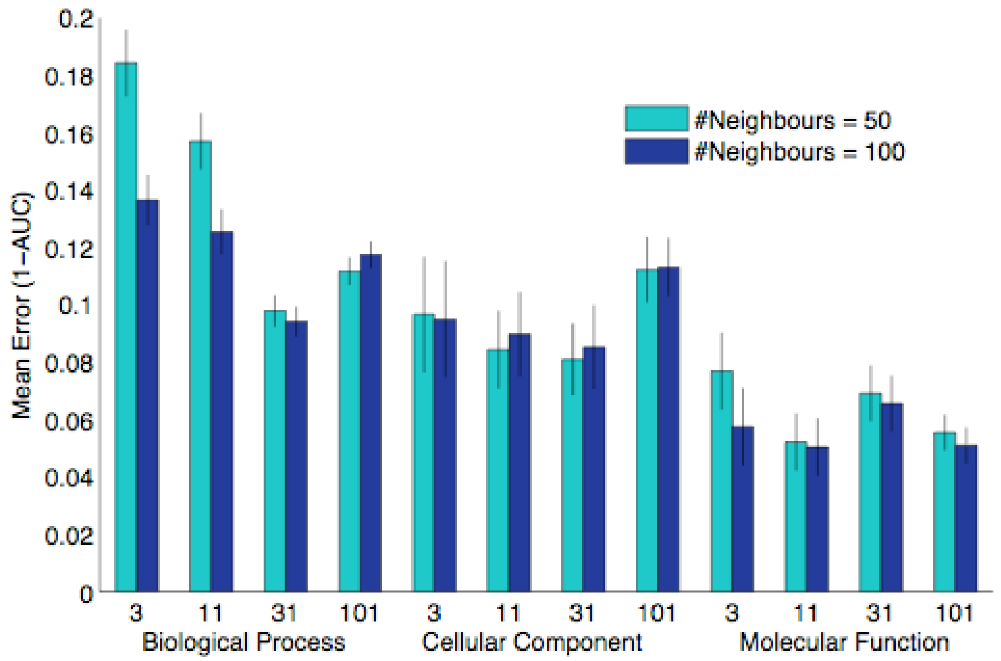

Figure S2. Effect of Network Sparsification on MouseFuncI Benchmark data. Bars demonstrate the prediction error in 12 evaluation categories based on test benchmark of MouseFuncI data with two sparsity levels. Performance is based on prediction of all categories on the test benchmark (1174 BP, 231 CC, and 442 MF categories). Error bars depict the standard error in prediction of all GO classes in each of the evaluation categories.

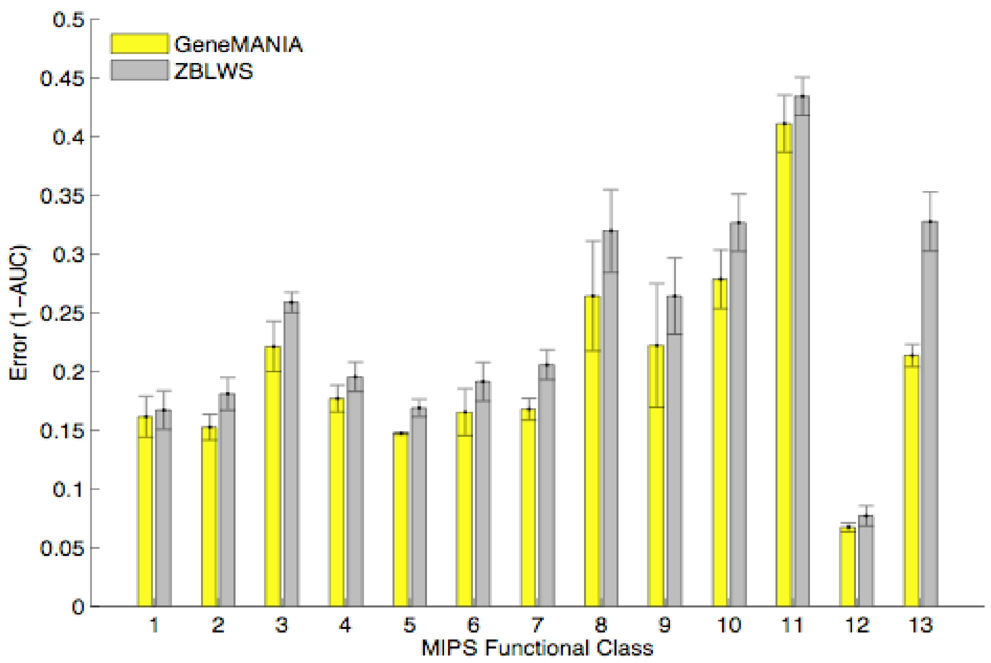

Figure S3. Effect of Label Bias on ROC Scores. Bars show the prediction error (measured with 1-AUC) for prediction of 13 functional classes from the five yeast benchmark data with a single association network (protein domains network) with GeneMANIA (k = average label) and ZBLWS (k = 0) . Error bars depict the standard error based five random split of 3-fold cross-validation trials (15 trials).

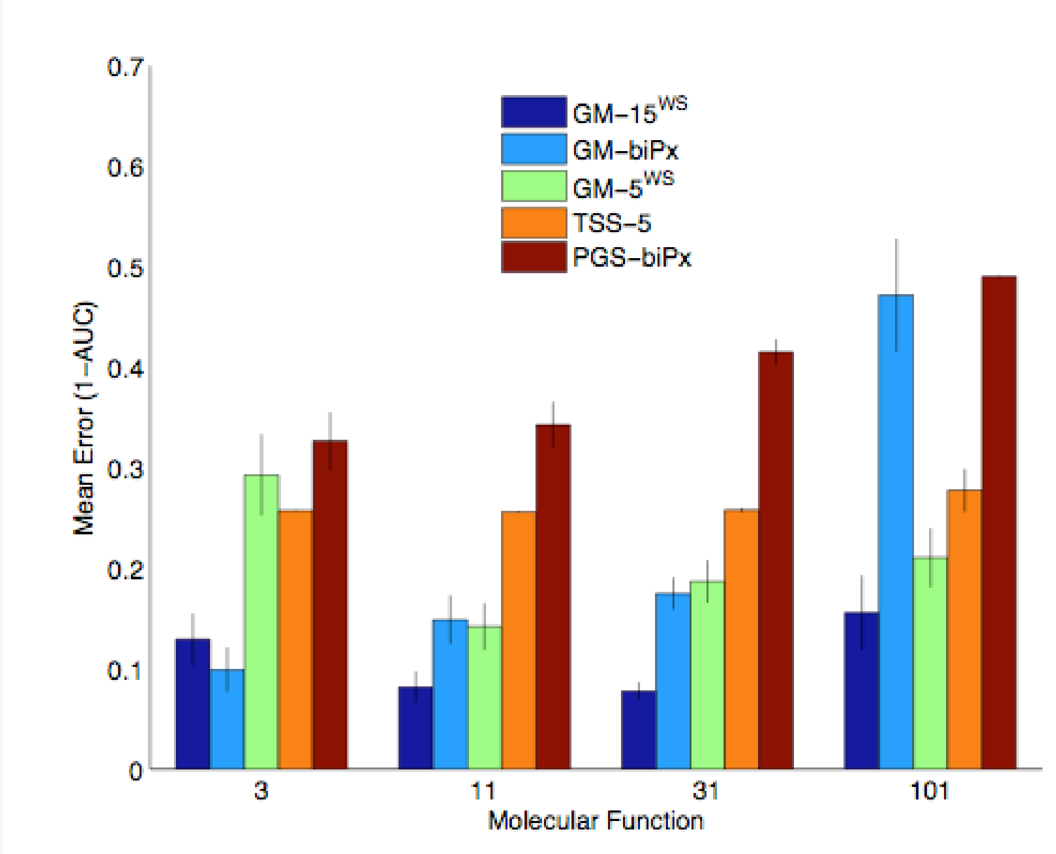

Figure S4. Prediction Performance of GeneMANIA, TSS, and PGS on functional classes from MF ontology. Bars show the prediction performance of GeneMANIA on the fifteen yeast networks (GM-15^{WS}), five yeast networks (GM-5^{WS}), the bioPIXIE network (GM-biPx), the TSS algorithm on the five yeast benchmark networks, and the Probabilistic Graph Search algorithm (PGS) (Myer's et al.) on the bioPIXIE network (PGS-biPx).

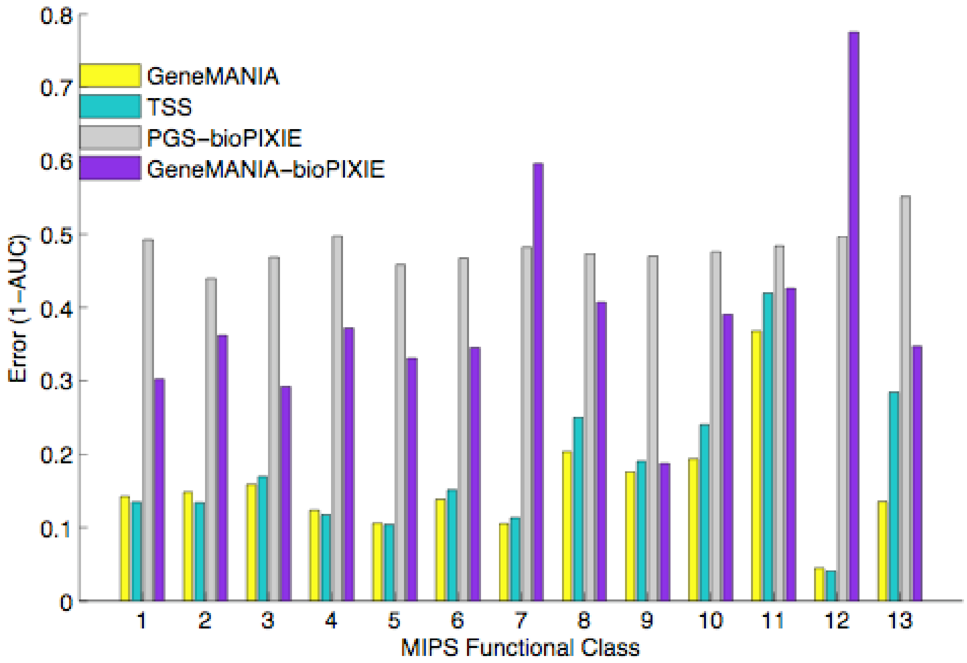

Figure S5. Prediction Performance of GeneMANIA on Yeast Benchmark Compared to bioPIXIE and the TSS algorithm. Prediction performance of GeneMANIA, TSS, and the Probabilistic Graph Search (PGS) algorithm (Myer's er al.). GeneMANIA and TSS depicts the performance of GeneMANIA with TSS on the five yeast benchmark networks. PGS-bioPIXIE and GeneMANIA-bioPIXIE depict the performance of GeneMANIA and PGS on the bioPIXIE network. The performance is evaluated using 3-fold cross-validation according to the 13 MIPS functional classes in the five yeast network benchmark.
